# Comparison of Droplet Digital PCR and qPCR for the Quantification of Shiga Toxin-Producing *Escherichia coli* in Bovine Feces

**DOI:** 10.3390/toxins8050157

**Published:** 2016-05-18

**Authors:** Bavo Verhaegen, Koen De Reu, Lieven De Zutter, Karen Verstraete, Marc Heyndrickx, Els Van Coillie

**Affiliations:** 1 Institute of Agriculture and Fishery Research (ILVO), Technology and Food Science Unit, Brusselsesteenweg 370, Melle 9090, Belgium; bavo.verhaegen@ilvo.vlaanderen.be (B.V.); koen.dereu@ilvo.vlaanderen.be (K.D.R.); karen.verstraete@ilvo.vlaanderen.be (K.V.); marc.heyndrickx@ilvo.vlaanderen.be (M.H.); 2 Department of Veterinary Public Health and Food Safety, Faculty of Veterinary Medicine, Ghent University, Salisburylaan 133, Merelbeke 9820, Belgium; lieven.dezutter@ugent.be; 3 Department of Pathology, Faculty of Veterinary Medicine, Bacteriology and Poultry Diseases, Ghent University; Salisburylaan 133, Merelbeke 9820, Belgium

**Keywords:** Shiga Toxin-producing *Escherichia coli*, real-time qualitative PCR, droplet digital PCR, PCR inhibition, cattle

## Abstract

Cattle are considered to be the main reservoir for Shiga toxin-producing *Escherichia coli* (STEC) and are often the direct or indirect source of STEC outbreaks in humans. Accurate measurement of the concentration of shed STEC in cattle feces could be a key answer to questions concerning transmission of STEC, contamination sources and efficiency of treatments at farm level. Infected animals can be identified and the contamination level quantified by real-time quantitative PCR (qPCR), which has its specific limitations. Droplet digital PCR (ddPCR) has been proposed as a method to overcome many of the drawbacks of qPCR. This end-point amplification PCR is capable of absolute quantification independent from any reference material and is less prone to PCR inhibition than qPCR. In this study, the qPCR-based protocol described by Verstraete *et al.* (2014) for Shiga toxin genes *stx*1 and *stx*2 and the intimin gene *eae* quantification was optimized for ddPCR analysis. The properties of ddPCR and qPCR using two different mastermixes (EMM: TaqMan^®^ Environmental Master Mix 2.0; UMM: TaqMan^®^ Universal PCR Master Mix) were evaluated, using standard curves and both artificial and natural contaminated cattle fecal samples. In addition, the susceptibility of these assays to PCR-inhibitors was investigated. Evaluation of the standard curves and both artificial and natural contaminated cattle fecal samples suggested a very good agreement between qPCR using EMM and ddPCR. Furthermore, similar sensitivities and no PCR inhibition were recorded for both assays. On the other hand, qPCR using UMM was clearly prone to PCR inhibition. In conclusion, the ddPCR technique shows potential for the accurate absolute quantification of STEC on the farms, without relying on standardized reference material.

## 1. Introduction

Shiga toxin-producing *Escherichia coli* (STEC), also known as verocytotoxin-producing *E. coli* (VTEC), remains a major foodborne pathogen of worldwide concern. STEC can be transmitted to humans through many different routes, either by direct contact with STEC contaminated fecal material, or indirectly via consumption of fecally contaminated meat, milk, fruits, vegetables or water [1,2]. Ruminants, especially cattle, are colonized by STEC and regarded as the natural reservoir [1]. STEC can be pathogenic to humans, causing mild to severe clinical symptoms [3]. *E. coli* O157:H7 remains the serotype which have been most frequently associated with severe symptoms, therefore most studies have examined the epidemiology of *E. coli* O157:H7 in cattle populations. However, the non-O157 STEC serogroups, such as O26, O103, O111 and O145, are increasingly being recognized and reported as important foodborne pathogens. Still, much less is known about these STEC serogroups [4,5]. The shedding pattern of STEC is mostly low in level, but can vary from 10 to 10^9^ CFU per gram feces, and is mostly short in duration [6]. However, some animals may be more persistent carriers of the pathogen or shed at higher levels (at least 10^4^ Colony Forming Units (CFU) per gram feces) for a longer period (>10 days) than others. These so-called “super-shedders” have a major impact on the on-farm prevalence and transmission, as well as in food contaminations [7,8]. The detection of these super-shedders is often performed using culture-based techniques to enumerate STEC in feces, such as direct plating, spiral plating and the most probable number (MPN) technique. These approaches ensure quantification of >10^2^ CFU/g feces, however the stressed and injured cells will not be counted [9]. Furthermore, the lack of an efficient selective isolation medium for all STEC strains makes these culture techniques too labor intensive to process large numbers of samples and even ineffective for various STEC strains [10]. A culture-independent method, such as quantitative polymerase chain reaction (qPCR), is often applied to quantify STEC in feces [11]. However, this method requires a DNA extraction and the limit of quantification is higher (10^3^–10^4^ CFU/g) compared to the culture-dependent techniques [6,9,12,13,14]. Furthermore, this approach is based on relative quantification and totally dependent on the accuracy of the standard curve construction [15]. Recently, a “third-generation PCR” or droplet digital PCR (ddPCR) has been developed. This technique allows for absolute quantification of target DNA molecules without the requirement for a standard curve. The technique is based on partitioning of the PCR sample into many thousands of droplets so that each contains one (or a few) or no copies of the target DNA. The absolute number of target DNA in the sample is calculated directly from the ratio of the positive to the total of partitions using binomial Poisson statistics [16]. The PCR amplification occurs in each droplet. The fluorescence signal of each droplet is individually counted. Since ddPCR is an end-point PCR, it is suggested to be more flexible concerning sample quality and, thus, less prone to PCR inhibition [17,18].

In this study, we optimized a qPCR protocol for the quantification of the main virulence genes of STEC for ddPCR use. Furthermore, we compared the sensitivity and resistance to PCR inhibition of both qPCR and ddPCR assays, using artificially and naturally contaminated cattle feces.

## 2. Results

### 2.1. Comparison of qPCR and ddPCR Standard Curves

Diluted series of gDNA of strains MB3936 and MB4378 were analyzed in order to compare qPCR using TaqMan^®^ Environmental Master Mix 2.0 (EMM; Life Technologies, Carlsbad, CA, USA) and ddPCR performance. Both assays exhibited an excellent degree of linearity (*R^2^*: 0.9959 to 0.9999). The slope values in qPCR ranged from −3.25 to −3.31, equivalent to 101% to 103% PCR efficiency. In ddPCR, the PCR efficiency ranged from 96% to 105%. Both assays were able to quantify the lowest tested gDNA concentration (2 × 10° target copies per µL). However, for ddPCR, reaction saturation was reached at a concentration of 2 × 10^5^ target copies/µL and therefore it was impossible to quantify this high concentration (Table 1).

The correlation between the qPCR-EMM and ddPCR measurements showed a degree of linearity (*R^2^*: 0.9899 to 0.9998) very close to 1. This suggests a very good correlation between qPCR and ddPCR assays.

### 2.2. Inhibition

**Figure 1:**
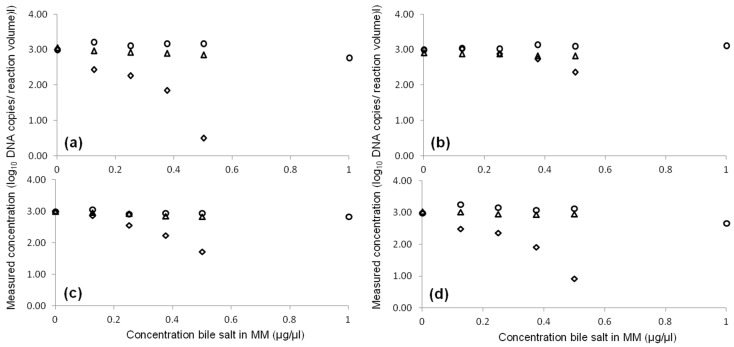
Influence of increasing concentrations of bile salt on the measured target DNA copies in qPCR-EMM (○), qPCR-UMM (◊) and ddPCR (∆) for *eae* (**a**), *stx*1 (**b**), *stx*2 (set a) (**c**) and *stx*2 (set b) (**d**) quantification. MM: mastermix.

Figure 1 shows the effect of an increasing amount of bile salts (0, 0.125, 0.25, 0.375, 0.5, 1 µg per µL reaction mixture) on qPCR-EMM, qPCR using TaqMan^®^ Universal PCR Master Mix (UMM; Life Technologies) and ddPCR. Concentration of bile salts up to 0.5 µg/µL in the PCR mixture did not affect the qPCR-EMM and ddPCR results. In contrast to qPCR-EMM, with ddPCR no measurements were possible with 1 µg/µL bile salts, because at this high concentration it was not possible to generate droplets. For qPCR-UMM, the amplification efficiency was substantially inhibited by increasing the concentration of bile salts. The PCR assays for *eae* and *stx*2 (set b) seemed to be more prone to PCR inhibition by bile salts in comparison to *stx*2 (set a) and *stx*1.

### 2.3. Artificial Contaminated Fecal Sample

**Figure 2:**
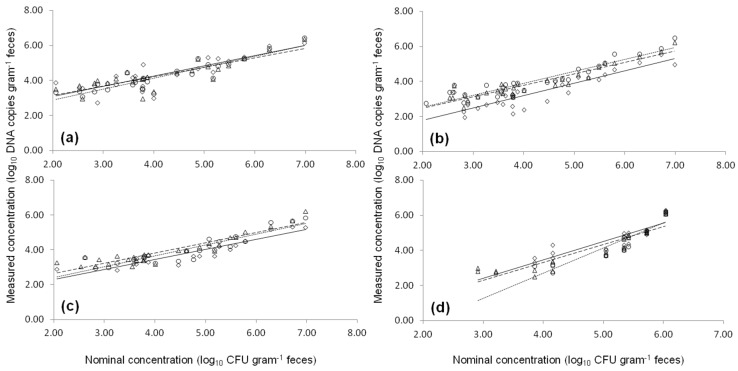
Quantification of *eae* (**a**), *stx*1 (**b**), *stx*2 (set a) (**c**), and *stx*2 (set b) (**d**) by qPCR-EMM (○,·····), qPCR-UMM (◊,──) and ddPCR (∆,─ ─) in cattle fecal samples artificially inoculated with STEC cells. Artificial inoculation was performed using various contamination levels of strain MB3936 (*stx*1, *stx*2 (set a) and *eae*) and MB4378 (*stx*2 (set b)).

Two STEC strains were separately added to *stx*- and *eae*-negative cattle feces at different concentrations, and inoculated samples were subjected to qPCR-EMM, qPCR-UMM and ddPCR analysis (Figure 2). The limit of quantification for both qPCR-EMM and ddPCR was 2.75 log copies g^−1^ feces for *stx*1 and *st*x2 (set b) and 3.06 log copies g^−1^ feces for *eae* and *stx*2 (set a). The results of the qPCR-UMM were generally lower compared to qPCR-EMM and ddPCR (*p* < 0.05), except for the *eae* assays and *stx*2 (set b) (Figure 2). However, the results of the Internal Amplification Control (IAC) showed no PCR inhibition in any of the samples.

**Figure 3:**
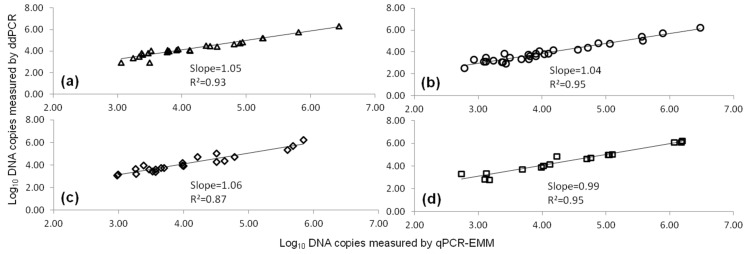
The linear correlation between qPCR-EMM and ddPCR measurements for *eae* (∆,**a**), *stx*1 (○,**b**), *stx*2 (set a) (◊,**c**) and *stx*2 (set b) (□,**d**) genes quantification in cattle fecal samples artificially inoculated with STEC cells. Artificial inoculation was performed using various contamination levels of strain MB3936 and MB4378.

The correlation between the qPCR-EMM and ddPCR measurements (>LOQ) showed *R*^2^ values ranged from 0.87 to 0.95 depending on the target gene (Figure 3). No significant differences were observed for the *eae* and *stx*2 (set b) assays by qPCR-EMM and ddPCR (*p* > 0.05). The *stx*1 qPCR-EMM measurement was significantly higher compared to ddPCR (*p* < 0.05), while the *stx*2 (set a) measurement was significantly lower (*p* < 0.05). However, the biological importance of these findings is less meaningful.

### 2.4. Natural Contaminated Fecal Samples

**Figure 4:**
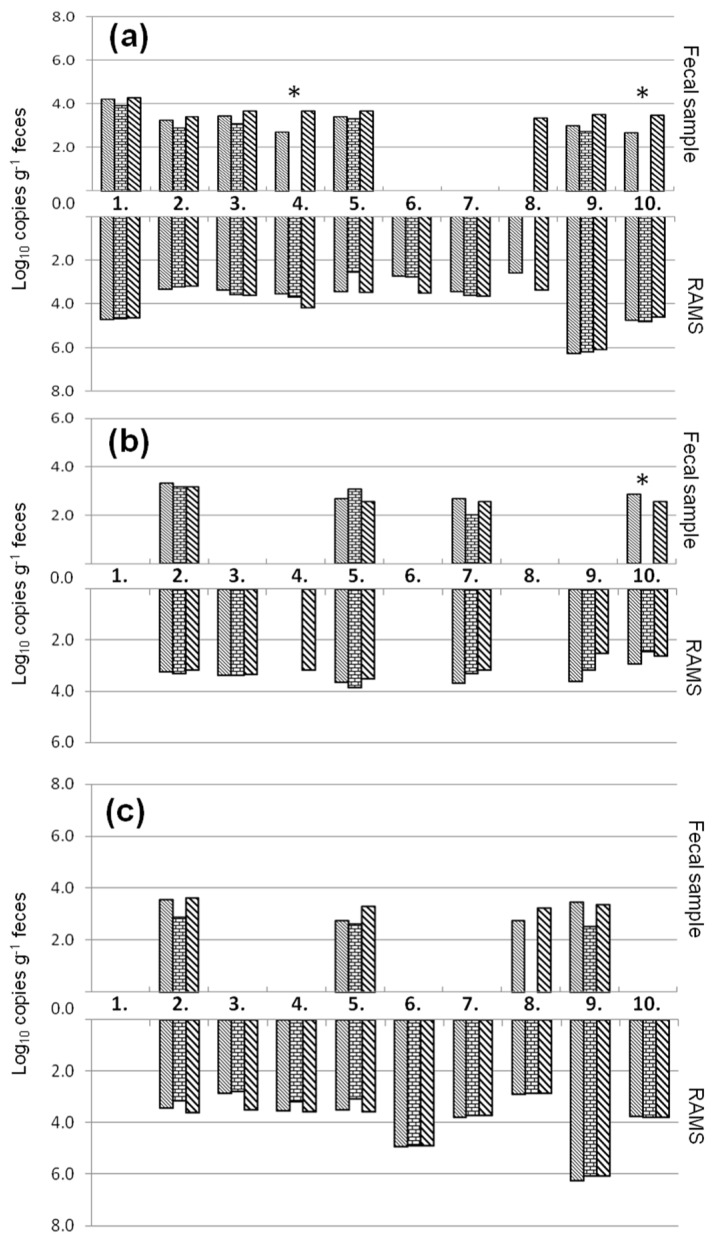
Quantification of *eae* (**a**), *stx*1 (**b**) and *stx*2 (**c**) genes by qPCR-EMM 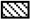, qPCR-UMM 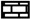 and ddPCR 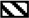 in cattle fecal samples and recto-anal-mucosal swabs (RAMS) of ten animals (1–10) of a STEC-positive farm. * Cattle fecal samples that showed PCR inhibition for the internal amplification control.

The number of target copies measured by qPCR-EMM, qPCR-UMM and ddPCR in fecal samples and recto-anal-mucosal swab (RAMS) of ten animals from a STEC-positive farm are presented in Figure 4. In all ten animals, *eae* was detected (7/10 fecal samples, 10/10 RAMS) with at least one of the three assays. *Stx*1 was found in seven animals (4/7 fecal samples, 7/7 RAMS) and *stx*2 in nine animals (4/9 fecal samples, 9/9 RAMS) with at least one of the three assays. In this experiment, a higher positive rate was noted from RAMS in comparison to fecal samples from the same animal. While the type of sample seemed important, no significant differences were observed between measurements by the qPCR-EMM and ddPCR assays above the limit of quantification (*p* > 0.05). The results of the IAC showed no PCR inhibition in any of the fecal samples or RAMS, except for fecal samples of animal 4 and 10 in the qPCR-UMM assay (Figure 4).

## 3. Discussion

This study evaluated ddPCR as a technique to quantify STEC in cattle feces in comparison to conventional qPCR. After some optimization of the ddPCR assay, by using a double quenched ZEN probe, and altering the primer/probe concentrations and annealing temperatures, our results showed that ddPCR had an excellent agreement with qPCR in DNA quantification. However, since 10^5^ target copies per µL template resulted in 100% saturation of positive droplets, the upper quantification limit for the ddPCR was notably lower in comparison to qPCR. Pinheiro *et al.* (2012) noted the same dynamic limit of 10^5^ target copies per µL template [18]. Therefore, the quantification of high levels of targets is a limitation of the ddPCR compared to the qPCR assay. However, in none of the ddPCR runs from naturally contaminated fecal samples in this study saturation were observed.

One of the main advantages of ddPCR compared to qPCR is that it would be less prone to inhibitors, which may be present in natural samples, even after DNA purification. These inhibitors may induce a shift in the amplification curve and therefore an increase in threshold cycle (Ct) during the qPCR. Since the ddPCR is an end-point PCR, the impact of such a shift would have much less influence on the final result. In this study indeed no PCR inhibition was observed with ddPCR, neither in the presence of bile salts added, nor in the tested natural samples. However, we observed that this disadvantage of the qPCR technique can be overcome by using a qPCR mastermix specially designed for matrices with high levels of inhibitors, such as TaqMan^®^ Environmental Master Mix 2.0 (EMM). In contrast, using TaqMan^®^ Universal PCR Master Mix (UMM) we clearly observed PCR inhibition, but the level was shown to be assay dependent. The latter corresponds to the results of Huggett *et al.* (2008) who demonstrated that the robustness of an assay has an important impact on the susceptibility of a PCR reaction to inhibitors [19]. Moreover, a suitable DNA purification step should prevent high levels of inhibitors, such as the bile salt concentrations used in this study.

Some studies reported an increased sensitivity of the ddPCR to detect low quantities of target DNA in comparison to qPCR [20,21,22]. Beer *et al.*, (2007) showed that low numbers of target copies in a droplet needed half of the number of cycles than the same assay conducted with a regular qPCR to reach the Ct. This reduction in required cycles could explain the higher sensitivity of the ddPCR assay [23]. However, in the present study similar LOQs were observed for both techniques. For the evaluation of qPCR and ddPCR using natural STEC contaminated samples both fecal and RAMS samples were investigated. More RAMS samples were found positive in comparison to the fecal samples of the same animals. Furthermore, PCR inhibition was observed in some of the fecal samples using qPCR-UMM, while in none of the RAMS samples inhibition was detected. The benefit of using RAMS instead of fecal samples was already demonstrated by Davis *et al.*, (2006) for the culture-dependent detection of STEC and has already been used for qPCR-based detection of STEC in cattle [9,24].

One of the main advantages of ddPCR is the accurate absolute quantification without the need to rely on a standard curve. This is an important advantage compared to qPCR because the construction of any standard curve requires accurately quantified template DNA, which might be difficult to obtain.

Despite the independence of a standard curve, the ddPCR is somewhat more time consuming and labor intensive compared to qPCR. Furthermore, the ddPCR platform is more expensive compared to qPCR. For qPCR in order to prepare, amplify and analyse 96 samples it took up to three hours, while ddPCR took up to 5.5 h. Furthermore, the cost per reaction was remarkably higher for ddPCR (ddPCR: ~€2.80, qPCR-EMM: ~€1.60, qPCR-UMM: ~€1.20). However, the need to include (different) standard curves for the quantification of eae, stx1, stx2 (set a) and stx2 (set b) using qPCR somewhat mitigates the difference in cost.

## 4. Conclusions

We have demonstrated that after some optimization efforts, accurate absolute quantification of the STEC target genes was possible with ddPCR. The same sensitivity compared to qPCR was observed, while ddPCR is independent of a standard curve. The accurate measurement of the concentration of shedded STEC in feces is a key answer for questions concerning transmission of STEC, contamination sources and efficiency of treatments at farm level. Because of the low sensitivity for inhibition, this technique shows promise for microbial detection and quantification in complex samples. The ddPCR technique shows potential, not only for the detection and quantification of STEC in the farms, but also as a valuable application in food safety in general.

## 5. Materials and Methods

### 5.1. Strains

Bacterial strains MB3936 (STEC O26; *stx*1+ *stx*2+ *eae*+) and MB4378 (STEC O138; *stx*2e+) were used in this study, both were isolated from humans. Both strains carry single copies of the tested genes. Both strains were stored at −80 °C using Pro-Lab Microbank cryovials (Pro-Lab, Richmond Hill, ON, Canada) according to the manufacturer’s instructions. Strains were cultured on Tryptone Soy Agar (TSA; Oxoid Ltd., Basingstoke, Hampshire, UK) and incubated at 37 °C for 24 h. A single colony from these culture plates was transferred into Tryptone Soy Broth (TSB; Oxoid). After incubation at 37 °C for 24 h the genomic DNA (gDNA) was purified using DNeasy Blood & Tissue kit (Qiagen Inc., Valencia, CA, USA) according to the manufacturer’s instruction for Gram-negative bacteria with an additional RNase step, and eluted in a final volume of 200 µL elution buffer. The concentration of the gDNA was measured using a Quantus^TM^ fluorometer (Promega, Madison, WI, USA). The following formula was used for calculation of the mass (*M*) of one genome:

$$M=n\times 1.096\times 10^{-21}\;\frac{gram}{bp}$$

For *E. coli* strain O157:H7 EDL933, the genome length (*n*) was determined as 5.53 × 10^6^ bp [25].

Both gDNA preparations were diluted in nuclease-free water (Qiagen) to 10^6^ copies/μL and stored as stock template at −20 °C until use.

### 5.2. qPCR Assays

qPCR for the quantification of the STEC virulence genes as described by Verstraete *et al.* (2014) was used [11]. In this assay, four different primer sets are used in singleplex, one primer set for the quantification of the subtypes of *stx*1, one for the quantification of *eae* and two primer sets for the quantification of the subtypes of *stx*2. Two master mix types were used, namely TaqMan^®^ Environmental Master Mix 2.0 (EMM) and TaqMan^®^ Universal PCR Master Mix (UMM). Each qPCR mixture (25 µL including 5 µL DNA template) contained: 1× PCR master mix (EMM or UMM), 300 nM of both F/R primers of the respective primer set and 100 nM of 5’-FAM labeled probe (Eurogentec, Seraing, Belgium) (Table 2). The thermal protocol was as follows: initial incubation at 95 °C for 5 min followed by 40 cycles of 95 °C for 15 s and 1 min annealing and elongation at 60 °C, and cooling at 40 °C for 30 s. For the use of UMM, which contains Uracil-DNA Glycosylase (UNG), an initial enzyme activation step was included at 50 °C for 2 min. A standard curve of a serial dilution of gDNA of STEC strain MB3936 for *eae*, *stx*1 and *stx*2 (set a) and of MB4378 for *stx*2 (set b, specific for *stx*2d and *stx*2e) was utilized. All qPCR assays were performed on a LightCycler^®^ 480 (Roche Diagnostics, Vilvoorde, Belgium) using the LightCycler 480 Software version 1.5.0 (Roche Diagnostics).

### 5.3. ddPCR Assays

Before the comparison of quantification of the STEC virulence genes using qPCR and ddPCR, the ddPCR assays were first optimized in two steps: optimization of (1) the thermal protocol and (2) the concentration and labeling of the primers and probes. For the optimization of the thermal protocol a range of annealing temperatures (55–65 °C) were compared. For the primers and probes different concentrations and the use of regular single-quenched probes *versus* double-quenched ZEN probes (Integrated DNA Technologies (IDT), Coralville, IA, USA) were evaluated. The ddPCR workflow and data analyses were performed according to the manufacturer’s instructions. Briefly, 20 µL of each reaction mixture was loaded into a sample well of an eight-channel disposable cartridge (Bio-Rad, Marnes-la-Coquettes, France) followed by 70 μL of droplet generator oil (Bio-Rad) into the oil-wells of the cartridge. Droplets were formed in the QX200 droplet generator (Bio-Rad). Droplets were then transferred to a 96-well PCR plate, heat-sealed with foil (Bio-Rad) in a PX1™ PCR Plate Sealer (Bio-Rad), and amplified with a T100 Touch Thermal Cycler (Bio-Rad). PCR reactions were analyzed with the QX200 droplet reader (Bio-Rad) and data analysis was performed using the QuantaSoft software (Version: 1.6.6., Bio-Rad). Note that a positive control is sufficient for the ddPCR technology, instead of the standard curve for the qPCR technology.

Satisfactory separation of positive and negative droplets for the target was achieved using the following optimized reaction mixture: 10 µL of 2× Supermix for Probe (No UTP) (Bio-Rad), 900 nM of both F/R primers, 250 nM of 5’-FAM labeled double-quenched probe in a mixture volume of 20 µL. Five µL of template DNA was added to each mixture. The optimized thermal protocol included an initial incubation step at 95 °C for 5 min followed by 40 cycles of a 3-step amplification at 95 °C for 15 s, 59 °C for 30 s, and 60 °C for 30 s, and cooling at 40 °C for 30 s. The threshold for a positive signal was set at a fluorescence amplitude of 1500. Only the reactions with more than 10,000 accepted droplets were used for analysis.

Once the conditions were optimized the dynamic range of both qPCR and ddPCR assay were compared. For this purpose, each stock template was serially diluted from 2 × 10^5^ to 2 × 10^0^ target copies per µL. For each PCR assay, three replicates were performed. The degree of linearity (*R^2^* value) and slope were calculated on the average numbers of target copies measured by qPCR and ddPCR assays.

### 5.4. Inhibition

Bile salt, a known PCR inhibitor present in faeces [26] was selected to evaluate the effect of PCR inhibition using qPCR with EMM (qPCR-EMM) and UMM (qPCR-UMM) and ddPCR. A concentration range (0, 0.125, 0.25, 0.375, 0.5, 1 µg Ox Bile Extract (Oxoid) per µL reaction mixture) was tested. This range of concentrations was tested based on a previous study which showed a change in signal output of the qPCR [26]. To each reaction mixture 10^3^ target copies were added. For each PCR assay, three replicates were performed.

### 5.5. Internal Amplification Control (IAC)

As internal amplification control a synthetic gene sequence inserted in a plasmid cloning vector synthesized by Integrated DNA Technologies (IDT) was used. The synthetic gene (5’-GAC GCA GTC TGT TGC AAG AG-TATATA-TAC CAG GCT ATT TTG CCT GCT TAT GTG C-TATATA-C AGC TGA AGC TTT ACG TTT TCG-3’) contained the primer/probe binding sites (underlined) for the *stx*1-forward-primer, the *eae*-probe and the *stx*1-reverse-primer. The concentration was measured using a Quantus^TM^ fluorometer (Promega), diluted in nuclease-free water to 10^2^ copies/μL and stored as stock IAC at −20 °C until use.

Five microliters of the stock IAC was added to each *stx*1 reaction. Additionally, 100 nM and 250 nM 5’-HEX labeled *eae*-probe for qPCR and ddPCR, respectively, was added for the detection of the IAC. The IAC at a concentration of 10^2^ copies/μL had no influence on the *stx*1 quantification. The comparison of the obtained results with the results of a parallel *stx*1 reaction containing the same amount of IAC, but in the absence of sample DNA enables the determination of the level of PCR inhibition. The IAC was included in all assays for the analyses of artificial and natural contaminated fecal samples.

### 5.6. Artificial STEC Contaminated Fecal Samples

Multiple cattle fecal samples were taken from a local combined (beef and dairy) farm. Eight samples that were found negative for the stx genes by qPCR assay, were used for artificial contamination. These fecal samples were subdivided in subsamples of 0.25 gram feces. Subsamples were inoculated with the appropriate volumes of diluted fresh overnight cultures of MB3936 or MB4378, to obtain 24 inoculation levels ranging between 1.2 × 10^2^ and 1.1 × 10^7^ CFU per gram feces. The artificially contaminated fecal samples (0.25 grams) were subjected to DNA extraction using the QIAmp DNA stool Mini kit (Qiagen). The extracted DNA was then analyzed in duplicate by both qPCR, using EMM and UMM, and ddPCR assays. The gene copy numbers in 1 g artificial contaminated feces were calculated while accounting for the dilution factor in the PCR assay (×160). The limit of quantification (LOQ) was defined as the lowest number of organisms that can be quantified [11]. The viable cell counts of the diluted cultures for artificial inoculation were determined by plating in triplicate onto Tryptone Soy Agar (TSA) using a spiral plater (Eddy Jet Spiral Plater, IUL instruments, Barcelona, Spain). After incubation of the plates at 37 °C for 24-h colonies were enumerated.

### 5.7. Natural STEC Contaminated Fecal Samples

Samples from ten animals were taken at a culture-confirmed STEC-positive local farm. From each animal two samples were collected. First, a recto-anal-mucosal swab (RAMS) was taken, using a sterile floqswab (Copan Diagnostics Inc., Murrieta, CA, USA), followed by a fecal sample taken directly from the rectum. Both samples were placed in plastic bags and transported on ice packs. Upon arrival in the laboratory 0.25 gram of all samples were subjected to DNA extraction using the QIAmp DNA stool Mini kit (Qiagen) and analyzed by both qPCR, using EMM and UMM, and ddPCR assays, as described above.

### 5.8. Statistical Analysis

All measured numbers of genomic copies were log-transformed prior to analyses. Significant differences between gene copy numbers measured by qPCR-EMM, qPCR-UMM and ddPCR assays were determined using the standard paired *t* tests. The significance level of all analyses was set at 0.05. The statistical analyses were performed with the software R [27].

## Figures and Tables

**Table 1:** ddPCR reaction saturation percentages of the standard curves for *eae*, *stx*1, *stx*2 (set a) and *stx*2 (set b) quantification.

Concentration (Copies per µL Template)	Gene			
	*eae*	*stx1*	*stx2* (Set a)	*stx2* (Set b)
2 × 10^5^	100%	100%	100%	100%
2 × 10^4^	87%	87%	87%	88%
2 × 10^3^	19%	19%	19%	21%
2 × 10^2^	2.03%	1.96%	2.06%	2.11%
2 × 10^1^	0.22%	0.26%	0.20%	0.18%
10^1^	0.12%	0.13%	0.13%	0.17%

**Table 2:** Overview of primers/probes sequences (all designed by [11]) and labels used in this study.

Gene	Primer or Probe *	Sequence (5′-3′)	Labeling (5′-3′)	
			qPCR	ddPCR
*eae*	*eae*-F	GGA AGC CAA AGC GCA CAA	-	-
	*eae*-R	GGC ICG AGC IGT CAC TTT ATA A	-	-
	*eae*-P	TAC CAG GCT ATT TTG CCI GCT TAT GTG C	FAM–BHQ-1	FAM–ZEN–IBFQ
*stx*1	*stx*1-F	GAC GCA GTC TGT IGC AAG AG	-	-
	*stx*1-R	CGA AAA CGI AAA GCT TCA GCT G	-	-
	*stx*1-P	ATG TTA CGG TTT GTT ACT GTG	FAM–MGBNFQ	FAM–ZEN–IBFQ
*stx*2	*stx*2-F	TCA GGC AIA TAC AGA GAG AAT TTC G	-	-
	*stx*2-R (set a)	CCG GIG TCA TCG TAT ACA CAG	-	-
	*stx*2-R (set b)	CCG GIG TCA TCG TAT AAA CAG	-	-
	*stx*2-P	CAC TGT CTG AAA CTG CT	FAM–MGBNFQ	FAM–ZEN–IBFQ
IAC	*stx*1-F	GAC GCA GTC TGT IGC AAG AG	-	-
	*stx*1-R	CGA AAA CGI AAA GCT TCA GCT G	-	-
	*eae*-P	TAC CAG GCT ATT TTG CCI GCT TAT GTG C	HEX–BHQ-1	HEX–ZEN–IBFQ

* Forward primers with suffix -F; Reverse primers with suffix -R; Probes with suffix –P; MGBNFB: minor groove-binding non-fluorescent quencher (Applied Biosystems), BHQ-1: black hole quencher (Eurogentec), ZEN-IBFQ: internal ZENTM fluorescence quencher- Iowa Black fluorescence quencher (IDT).

## Abbreviations

The following abbreviations are used in this manuscript:

qPCR: real-time quantitative PCR
ddPCR: Droplet digital PCR
EMM: TaqMan^®^ Environmental Master Mix 2.0
UMM: TaqMan^®^ Universal PCR Master Mix
STEC: Shiga toxin-producing *Escherichia coli*
VTEC: Verocytotoxin-producing *Escherichia coli*
*R^2^*: degree of linearity
IAC: Internal Amplification Control
TSB: Tryptone Soy Broth
UNG: Uracil-DNA Glycosylase
RAMS: recto-anal-mucosal swab
LOQ: limit of quantification
